# Exploring the Roles of Dietary Herbal Essential Oils in Aquaculture: A Review

**DOI:** 10.3390/ani12070823

**Published:** 2022-03-24

**Authors:** Mahmoud A. O. Dawood, Mohammed F. El Basuini, Sevdan Yilmaz, Hany M. R. Abdel-Latif, Mahmoud Alagawany, Zulhisyam Abdul Kari, Mohammad Khairul Azhar Abdul Razab, Noor Khalidah Abdul Hamid, Tossapol Moonmanee, Hien Van Doan

**Affiliations:** 1 Animal Production Department, Faculty of Agriculture, Kafrelsheikh University, Kafr El-Sheikh 33516, Egypt; 2 The Center for Applied Research on the Environment and Sustainability, The American University in Cairo, Cairo 11835, Egypt; 3 Animal Production Department, Faculty of Agriculture, Tanta University, Tanta 31527, Egypt; m_fouad_islam@yahoo.com; 4 Department of Aquaculture, Faculty of Marine Sciences and Technology, Canakkale Onsekiz Mart University, Canakkale 17100, Turkey; sevdanyilmaz@comu.edu.tr; 5 Department of Poultry and Fish Diseases, Faculty of Veterinary Medicine, Alexandria University, Alexandria 22758, Egypt; hmhany@alexu.edu.eg; 6 Poultry Department, Faculty of Agriculture, Zagazig University, Zagazig 44511, Egypt; dr.mahmoud.alagwany@gmail.com; 7 Department of Agricultural Sciences, Faculty of Agro-Based Industry, Universiti Malaysia Kelantan, Jeli Campus, Jeli 17600, Malaysia; 8 School of Health Sciences, Universiti Sains Malaysia, Health Campus, Kubang Kerian 16150, Malaysia; khairul.azhar@usm.my; 9 School of Biological Sciences, Universiti Sains Malaysia, Minden, Pulau Pinang 11800, Malaysia; 10 Department of Animal and Aquatic Sciences, Faculty of Agriculture, Chiang Mai University, Chiang Mai 50200, Thailand; tossapol.m@cmu.ac.th (T.M.); hien.d@cmu.ac.th (H.V.D.); 11 Innovative Agriculture Research Center, Faculty of Agriculture, Chiang Mai University, Chiang Mai 50200, Thailand

**Keywords:** alternative medication, herbs, aquaculture, antioxidants, immunity, essential oils

## Abstract

**Simple Summary:**

Essential oils, also known as aetheroleum, have a variety of therapeutic properties, including analgesic, adaptogen, and astringents, among others. Essential oils have potential growth-promoting, antibacterial, and immunostimulant effects for several fish species. Furthermore, they potentiate the antioxidative capacity and the resistance of aquatic animals against infectious diseases. This article spotlights on the essential oils derived from selected medicinal plants, and their roles in the improvement of the performances of aquatic animals.

**Abstract:**

The aquaculture sector is one of the main activities contributing to food security for humanity around the globe. However, aquatic animals are susceptible to several farming stressors involved in deteriorated growth performance, reduced productivity, and eventually high mortality rates. In some countries still, antibiotics and chemotherapies are comprehensively applied to control biotic stressors. Aside from the apparent benefits, the continuous usage of antibiotics develops bacterial resistance, deteriorates bacterial populations, and accumulates these compounds in the aquatic environment. Alternatively, environmentally friendly additives were used to avoid the direct and indirect impacts on the aquatic ecosystem and human health. In aquaculture, medicinal herbs and extracts are extensively used and approved for their growth-promoting, anti-inflammatory, and antioxidative properties. Herbal essential oils contain many bioactive components with powerful antibacterial, antioxidative, and immunostimulant potentials, suggesting their application for aquatic animals. Essential oils can be provided via diet and can benefit aquatic animals by improving their well-being and health status. The use of essential oils in aquafeed has been studied in a variety of aquatic animals to determine their beneficial roles and optimum doses. The outputs illustrated that herbal essential oils are exciting alternatives to antibiotics with prominent growth promotion, antioxidative, and immunostimulant roles. Herein, we reviewed the beneficial roles of essential oils in aquaculture. This review also aims to describe trends in herbal essential oils use, mainly in commercial fish species, and to analyze different factors that affect essential oils’ efficacy on the growth performance, antioxidative, and immune responses of finfish species.

## 1. Introduction

The expansion of the population requires sustainable and safe food resources. The aquaculture sector provides humanity with secure food and profitable income [1]. Nevertheless, the aquaculture sector is confronted with serious challenges related to immunocompromised, deteriorating health and well-being, substantially resulting in a high mortality of farmed aquatic animals [2]. Intensive and super-intensive farming practices induce stress to the aquatic animals, thereby increasing the possibility of infection with pathogenic invaders [3]. Synthetic antibiotics and chemotherapies are commonly used to relieve the negative impacts of infection, and enhance the immunological response and overall well-being of aquatic animals. The continuous application of antibiotics has resulted in several negative impacts on aquatic animals (e.g., bacterial resistance developed against antibiotics, suppressed host immunity, imbalance of microbial populations, and hazardous environmental features) [4]. Accordingly, safer alternative methods, such as organic feed additives, are highly suggested to mitigate and control pathogenic infections in aquatic animals [5]. Feed additive inclusion is an appropriate strategy that can be used in aquafeed at specific doses and can be orally administered to aquatic animals [6]. Aquafeed is commonly supplemented with various additives such as probiotics, prebiotics, and herbal substances to stimulate the health and well-being of aquatic animals [7,8]. Specifically, the inclusion of medicinal herbs and their extracts in aquafeed is involved in multiple functional roles associated with active metabolites and functional components [9].

Medicinal herbs, also known as phytobiotics, and the essence and oil derivatives of these plants, play significant roles as appetite enhancers, growth promoters, and immunostimulators for aquatic animals [10]. Essential oils (EOs) have gained special focus as natural antioxidant and immunostimulant agents. The chemical structural compound of EOs is resistant to gastric acid, ensuring its efficacy and effect [11,12]. Furthermore, these volatile oils enhance palatability and regulate appetite control genes in the hypothalamic−pituitary glands of fish [11]. EOs show a strong antibacterial effect against pathogenic bacteria by impairing their activity and damaging the bacterial cell walls [13]. Then, beneficial bacteria play influential roles in food digestion mediated by the secretion of digestive enzymes, along with improved local intestinal immunity and high resistance to the challenged pathogens [14]. In addition, EOs enhance the permeability of intestinal barriers and increase intestinal nutrient absorption. The immune system-boosting effects of EOs are attributed to the increase in intestinal immunity [3]. The balanced intestinal microbiota (increased abundance of beneficial microorganisms and decreased abundance of pathogenic microorganisms), the inhibition of pathogenic bacteria’s adhesion sites, and the modification of the intestinal pH are the primary actions of EOs to relieve the pathogenic effects of bacteria on aquatic animals [15,16]. In fish challenged with pathogenic bacteria, EOs have been shown to improve the survival rate of many species [17,18,19,20,21,22]. Further, EOs contain high concentrations of polyphenols and natural antioxidants that remove the free radicals that cause lipid peroxidation and immune cell damage [23,24].

The ultimate beneficial effects of herbal EOs on fish and their mechanisms of action are not well described. This review aims to illustrate the effects of EOs and their modes of action on fish and to highlight knowledge gaps for future studies.

## 2. Natural Sources of Essential Oils

Essential oils (EOs) are a lipophilic mixture of organic compounds from the secondary metabolism of aromatic plants, which mostly are limpid liquid (colorless) with an obvious fragrance [25,26]. Plant EO constituents include two main groups of terpene-derived compounds: the first consists of hydrocarbon terpenes/terpenoids, and the second group includes oxygenated molecules, which in hydrocarbon terpenes derivatives (e.g., alcohols, aldehydes, ketones, phenols, acids, and esters) represent the common form, while phenylpropanoids and their derivatives are another class of oxygenated compounds. Rarely, one or more sulfur or nitrogen molecules are found in a few compounds of EOs [26,27]. EOs may contain dozens of ingredients in a trace proportion of the wet weight of the plant origin source [26]. In most cases, EOs are distinguished by two or three major components in relatively high ratios (major constituents of 20–95%) compared with other components that occur in small amounts (secondary constituents of 1–20% and trace components of below 1%) [28,29]. The chemical composition of EOs may vary according to plant species, the development stage, cultivation and environmental condition (i.e., soil and climate) [30], oil extraction, and processing methods [31,32]. Hence, various types of EOs are extracted, and their chemical composition and organoleptic properties are verified as follows.

### 2.1. Menthol

Menthol (mint camphor) is a covalent organic cyclic monoterpene alcohol (C_10_H_20_O: 2-Isopropyl-5-methyl-cyclohexanol) that exists in eight optically active isomers with different organoleptic properties from four stereoisomers (menthol, isomenthol, neomenthol, and neoisomenthol) in two optic forms (levogirous and dextrogirous) [33]. Menthol is a colorless or white waxy crystalline solid substance at room temperature that melts slightly above it, and was first isolated in 1771 by the Dutch botanist Gambius [34]. The L- or (−)-menthol from the natural sources (plant origin) or synthesized is the most stable and preferred isomer [35]. Natural menthol is primarily derived from aromatic plants as it is the main constituent of EOs of the genus *Mentha* sp. [36]. *Mentha arvensis* L., (syn. *M. canadensis* L., Japanese mint) cornmint-MA, and *M. piperita* L. (Hudson) peppermint-MP are two well-known menthol mints in cultivation [35]. Menthol represents 70–90% of cornmint oil and about 20–60% peppermint oil [37,38]. The main supply of the world’s menthol production (19,170 tones) is obtained naturally (67.14%) exclusively from *M. canadensis,* and about 6300 tones (32.86%) are produced synthetically [39].

### 2.2. Linalool

Linalool is an odorant non-cyclic monoterpene alcohol (C_10_H_18_O: 3,7-Dimethyl-1,6-octadien-3-ol) found in nature in two stereoisomers: Licareol (R)- or (−)-linalool, and Coriandrol (S)- or (+)-linalool [40,41]. Linalool represents up to 90.6% of the oil constituents of over 200 aromatic plant species of different families (Supplementary Materials, Table S1) [38].

### 2.3. Myrcene

Myrcene is an alkene natural hydrocarbon acyclic monoterpene compound (C_10_H_16_: 7-Methyl-3-methylene-octa-1,6-diene) that exists in two isomers, the first of which is β-myrcene (the natural form), and the second form (α-myrcene) is not available naturally, but can be prepared industrially [42]. Myrcene is a colorless liquid found naturally in different sources (Supplementary Materials, Table S2) [38,43,44,45].

### 2.4. Eucalyptol

Eucalyptol is a cyclic monoterpenoid ether alcohol (C_10_H_18_O: 1,3,3-Trimethyl-2-oxabicyclo 2.2.2 octane) that is a colorless liquid that exists plentifully in nature [46]. Eucalyptol, also known as 1,8-cineole, and its isomer (1,4-cineole), occur naturally in the same plant species, although at much lower concentrations of 1,4-cineole than 1,8-cineole [47]. Eucalyptol is the main component (up to 80%) of EOs of Eucalyptus leaves (*Eucalyptus* spp.) [48,49]. It is present in varying proportions in the essential oils of some other aromatic plants (Supplementary Materials, Table S3) [38].

### 2.5. Globulol (Ledol)

Globulol is known as 5,10-cycloaromadendrane sesquiterpenoids alcohol (C_15_H_26_O: 1,1,4,7-tetramethyl-2,3,4a,5,6,7,7a,7b-octahydro-1aH-cyclopropa-e-azulen-4-ol), and exhibits a potent antimicrobial effect [50]. Globulol is also found in varying concentrations in EOs of various plants that belong to different families such as Myrtaceae, including *Eucalyptus* spp., with a rate of 5.3% extracted from the leaves and white kunzea (*Kunzea ambigua* (Sm.) Druce) by 11.2% from leaf oil; the family of Asteraceae, including Vassoura (*Baccharis dracunculifolia* DC), with a rate of 2.5–14.5% from leaf oil; the family of Cyperaceae such as Piri-piri (*Cyperus articulatus* L.) by 3.2–4.6% from rhizomes oil; and the family of Valerianaceae, e.g., Valerian (*Valeriana officinalis* L.) at a rate of 2.1% from root oil [38,50,51].

### 2.6. Spathulenol

Spathulenol is a viscous colorless tricyclic sesquiterpene alcohol with antileishmanial impacts (C_15_H_24_O: 1aR, 4aR, 7S, 7aR, 7bR)–1, 1, 7-Trimethy l-4-methylidene-1a, 2, 3, 4a, 5, 6, 7a, 7b-octahydrocyclopropa -h- azulen-7-ol) that is a major constituent of EOs extracted from the fresh leaves of croton species (*C. argyrophylloides*, *C. jacobinensis*, and *C. sincorensis*) at 42.54%, 15.41%, and 9.58%, respectively [51]. It occurs as the main component in the oil originating from the male and female specimen leaves of *B. semiserrata* DC, achieving 50.75 and 42.65%, respectively [52]. Among other sources, the oil is extracted from the leaves of *E. polybractea* R. T. Baker (14.3%), *B. dracunculifolia* DC (2.6–10.0%), *Pilocarpus jaborandi* Holmes, and *P. microphyllus* Stapf. (7.6%) [38]. Spathulenol is one of the main constituents of leaf EOs of *Myrciaria tenella* (DC.) Berg (9.7%) [53]. In addition, it is the main component (20.7%) of the essential oil of air-dried herb of *Origanum vulgare* L. ssp. [54].

### 2.7. Guaiol (Champacol)

Guaiol is a sesquiterpenoid alcohol (C_15_H_26_O: 2-(3S,5R,8S)-3,8-Dimethyl-1,2,3,4,5,6,7,8-octahydro-5-azulenyl-2-propanol) that has anti-cancer, anti-anxiety, anti-inflammatory, anti-bacterial, and antioxidant properties [53,55,56,57]. Guaiol is one of many terpenes found in the oil of several aromatic plants, especially oils of a wood origin, e.g., guaiacwood (*Bulnesia sarmientoi* Lorentz ex Griseb.) at 26.8%, cypress emerald (*Callitris columellaris* F. Muell.) at 20.0%, cypress jade (*C. glaucophylla* Joy Thomps. and L.A.S.F. Muell.) at 14.7%, cypress blue (*C. intratropica* R.T. Baker and H.B. Sm.) at 13.7%, and araucaria (*Neocallitropsis pancheri* (Carriere) de Laub.) at 6% [38]. In addition, guaiol represents 13.1% of the leaf oil constituent of *Calycorectes sellowianus* O. Berg [53].

### 2.8. Caryophyllene Oxide

Caryophyllene oxide (β-caryophyllene) is the oxidized form of caryophyllene (C_15_H_24_O: 1R, 4R, 6R, 10S-4, 12, 12-trimethyl-9-methylidene-5-oxatricyclo-8.2.0.0 (4,6)-dodecane) with therapeutic applications [45]. There is a sesquiterpenoid compound called caryophyllene found in the essential oils of common eucalyptus Melaleuca stypheloides in concentrations as high as 43.8% [58]. According to Tisserand and Young [38], β-caryophyllene is one of the major terpenes found in EOs originating from various plant species (Supplementary Materials, Table S4).

### 2.9. Thymol

Thymol is a monoterpenoid phenolic compound (C_10_H_14_O: 5-Methyl-2-(propan-2-yl)phenol) that exists naturally along with its isomer carvacrol [59,60]. Thymol occurs in varying percentages in the EOs of thyme species leaves (*T. zygis* (30.9–74.0%), *T. vulgaris* (48.3–62.5%), *T. serpyllum* L. (16.7–25.9%), *T. zygis* (25.5%), and *T. satureioides* Coss. and Bal. (10.0%); dried aerial parts of oregano flowering plant (*Lippia graveolens*) HBK (60.6 %)) and ajowan seeds (*Trachyspermum ammi* L. (36.9–53.8%) [38].

### 2.10. Carvacrol (CVC)

Carvacrol (cymophenol) is a natural monoterpenoid phenol (C_10_H_14_O: 2-Methyl-5-(propan-2-yl) phenol) that occurs along with its isomer thymol [46]. Carvacrol exhibits a distinct set of biological activities including antioxidant, antitumor, antibacterial, antifungal, and insecticidal properties [25,61]. Carvacrol is the primary compound of EO constituents of Lamiaceae species, including oils from aerial parts of oregano plants, including *O. onites* (66.5–80.4%), *O. majorana* L. (23.3–81.0%), *O. vulgare* (61.6–83.4%), *L. graveolens* HBK (0.5–24.8%), savory (*Satureia hortensis* L.) (43.6–70.7%), and *S. montana* L. (46.5–75.0%), thyme (*Thymbra spicata* L.) (70.0%), *Thymus vulgaris* L. (20.5%), and *T. satureioides* Coss. and Bal. Aerial parts (20.0%) [38,46].

### 2.11. Terpinen-4-ol

Terpinen-4-ol is a natural monoterpene isomer of terpineol (C_10_H_18_O: 4-methyl-1-propan-2-ylcyclohex-3-en-1-ol) [62] that is a promising potent therapeutic agent as it has antiviral, bactericidal, antifungal, anti-tumoral, anti-inflammatory, analgesic, insecticidal, and acaricidal activities [63,64,65,66]. Terpinen-4-ol is the primary component (30–48%) of tea tree oil (*M. alternifolia*), originating from the leaves [38,67]. Terpinen-4-ol occurs in varying percentages in EOs of plairhizomes (*Zingiber montanum* Theilade: 41.7%), marjoram freshly dried flowering plant (*O. majorana* L.: 16.4–31.6%), basil leaves (*Ocimum canum* Sims.: 7.5–26.8%), Kewda flowers (*Pandanus fascicularis* Lam.: 0–21.0%), sugandh mantri rhizomes (*Homalomena aromatica* Schott.: 17.2%), juniper berries (*Juniperus communis* L.: 1.5–17.0%), mace pericarp (*Myristica fragrans* Houtt.: 4.4–14.0%), and nutmeg kernels (*M. fragrans* Houtt.: 1.0–10.9%) [38].

### 2.12. Dehydrofukinone

Dehydrofukinone (DHF) or 9,10-Dehydrofukinone is a sesquiterpene ketonic compound (C_15_H_22_O: (4aR,5S)-4a,5-dimethyl-3-propan-2-ylidene-5,6,7,8-tetrahydro-4H-naphthalen-2-one) that possesses sedative properties [68,69,70]. DHF represents the main component (22%) of canela-amarela leaf essential oil (*Nectandra grandiflora* Nees) [69]. In addition, DHF isolated from the aerial parts of *Senecio* spp. (*S. punae*, *S. humillimus*, *S. aureus*, and *S. viridis*) shows a high antifungal activity (92.7 ± 0.2%) [71] and has a beneficial effect on non-pathogenic bacteria (*L. plantarum*) [72].

## 3. Effects of Essential Oils on Growth and Gut Bacterial Communities

Eco-friendly natural alternatives to antibiotics as growth stimulators in aquaculture are very trendy [73,74]. The compounds of essential oils are an area of interest in many perspectives due to their distinctive biological properties [75]. Botanical products, including essential oils, have been shown to improve a variety of biological activities in aquatic animals, including growth, appetite stimulation, anesthetic, anti-stress, antimicrobial, tonic, and immunomodulatory effects [76,77,78,79,80,81]. The biological properties of EOs are determined by their major bioactive constituents with their additive or/and synergistic effects with each other or/and with the biological system at a cellular level or below it (electron flow) [82,83,84,85]. The positive aspects of EOs on growth are similar to the effects of prebiotic (prebiotic-like effect) and can be linked to intestine morphological and physiological changes, as well as to modulation of the gut microbiota [75,86].

Bacterial communities are influenced by environmental, nutritional, microbiological, and genetic factors [87,88,89,90]. Under normal conditions, the microbiota of GIT surfaces contains a dynamic microbial equilibrium of pathogenic and saprophytic bacteria [87,91]. The maintenance of a healthy GIT microbiota has an impact on the host body’s performance and activities, such as nutrient utilization, digestibility, and immune modulation, because it can modulate the gene expression involved in epithelial proliferation, nutrient metabolism, and immune responses, as well as prevent the development of intestinal disorders and disrupting intestinal homeostasis [87,92,93,94]. The main role of EOs in modifying the gut microflora is the inhibition of pathogenic (harmful) bacterial groups and providing the opportunity for other groups (beneficial microflora) to dominate the gut [92,93]. The indirect effects of EOs on the intestinal microbiota can occur through changes in the intestinal environment, including changes in pH, and the type and amount of secretions of the intestinal mucosa [12,79,94,95,96,97]. The hydrophobicity of EOs is markedly affected by the pH value, which will control their antibacterial effect on the bacterial cell membrane. In this regard, rainbow trout (*Oncorhynchus mykiss*) provided with dietary *Thymus vulgaris* EO showed a marked antibacterial response against *Vibrio anguillarum* in the GIT [98]. Furthermore, Zhang et al. [99] reported that common carp (*Cyprinus carpio*) treated with Origanum EO had an increased count of *Propionibacterium*, *Brevinema*, and *Corynebacterium*_1, while decreasing *Vibrio* genera. Nile tilapia (*Oreochromis niloticus*) fed diets supplemented with essential oil from lemongrass (*Cymbopogon citratus*) and geranium (*Pelargonium graveolens*) had decreased counts of total bacteria, coliforms, *Escherichia coli,* and *Aeromonas* spp. in their intestine [100].

Reports of the effect of EOs on the intestinal microflora of aquatic organisms are scarce. In this respect, Giannenas et al. [101] assessed the dietary supplementation with carvacrol or thymol derived from *T. vulgaris* EO on rainbow trout intestinal microbiota. These authors showed a significant modulation in the gut microbiota characterized by a reduction of the total anaerobic bacteria. The efficacy of carvacrol and thymol incorporation in the diet for 6 weeks was also observed as modulating the intestinal microflora in red hybrid tilapia (*O. niloticus* × *O. aureus*) [102]. In contrast, no significant impacts of EOs on the intestinal microflora were reported in red drum (*Sciaenops ocellatus*) fed diets enriched with *O. americanum* EO [97] and in rainbow trout fed a diet supplemented with *T. vulgaris* EO [98]. Nonetheless, Zhang et al. [99] showed that oregano EO could alter the intestinal microbiota of the koi carp intestine by increasing the bacterial communities of Propionibacterium, Brevinema, and Corynebacterium.

The direct and indirect impacts of EOs on the gut microbiota enhance nutrient digestion and absorption, which positively affect fish growth by increasing amino acid array for protein synthesis and through deposition in the musculature [75]. In addition, EOs can directly enhance the appetite, digestion, absorption, anti-inflammatory, and antioxidant activities through maintaining intestinal health [103,104,105]. Maintaining a healthy intestine supports the continuity of its vital role in digestion and absorption, which significantly affects growth [106]. The improvement indicator of intestine health is an increase in the absorption area due to an increase in the intestinal secretion sources (submucosal tissues, goblet cells and crypt, and tunica muscularis), free of inflammatory and/or degenerative alterations, as well as the villus number, height, and width [107,108]. In this regard, dietary oregano EOs positively impact the intestine histomorphometry of the common carp fingerlings, including an improvement in the morphological structure of the intestinal villus [108]. Likewise, thymol dietary incorporation increased the length of the intestinal villus in Nile tilapia [109]. Moreover, Ferreira et al. [110] concluded that dietary oregano EO increased the intestinal absorptive area of yellow tail tetra fish alongside a significant glycogen accumulation in the liver. Furthermore, the positive influences of EOs on the growth performance may be due to the increased secretion and activity of GIT protease, amylase, and lipase, as reported by Zhang et al. [99].

The impacts of the dietary supplement of EOs on fish growth performance is described in Table 1. The growth of Nile tilapia responded in a positive way with the dietary supplementation of oregano EO [111,112], cinnamaldehyde and thymol [113], limonene and thymol [114], clove basil EO [115], encapsulated oregano oil containing 7.5% of carvacrol, and 2.5% of thymol (Silaacid^®^) [116]. Likewise, Hassaan and Soltan [117] found that EOs of fennel and garlic, alone or in combination with *Bacillus licheniformis*, had a positive effect on Nile tilapia fry growth performance and feed efficiency. In the case of silver catfish (*Rhamdia quelen*), lemon verbena (*Aloysia triphylla*) EO has been shown to promote better growth [118]. In the case of Mozambique tilapia (*O. mossambicus*), curcumin (*Curcuma longa*) EO upregulated the mRNA expression of the growth factor (IGF-1 and IGF-2) genes in the muscle [119]. In addition, sweet orange peel (*Citrus sinensis*) EO [17] and lemon peel (*C. limon*) EO [18] improved the growth performance of *O. mossambicus*.

Sönmez, et al. [120] demonstrated a favorable growth performance and feed efficiency of rainbow trout juveniles who consumed diets containing sage (*Salvia officinalis*) and thyme (*T. vulgaris*) oils, and the lowest performance was found with mint oil (*M. spicata*) supplementation. Oregano EO (*O. heracleoticum* L.) with its main constituents (carvacrol and thymol) exhibited affirmative growth in common carp [20], great sturgeon (*Huso huso*) [121], yellowtail tetra (*Astyanax altiparanae*) [122], rainbow trout [22,123], and channel catfish (*Ictalurus punctatus*) [21]. Carvacrol- and thymol-based diet supplements had a positive influence on trout growth and feed utilization, similar to Nile tilapia [124]. In addition, Giannenas et al. [101] confirmed that the use of carvacrol or thymol extracted from *T. vulgaris* as a dietary supplement improved the growth of rainbow trout. Gonçalves et al. [125] reported an improvement in the intestinal villus, nutrient utilization, and the growth of European sea bass (*Dicentrarchus labrax*) with dietary inclusion of a commercial EO product (Biomin^®^ Digestarom PEP MGE 150).

## 4. Essential Oils as Natural Antioxidants

Oxygen reactive species (ROS) are pro-oxidant compounds that generate by the partial reduction of oxygen in the mitochondria during the oxidative metabolism as second messengers for various growth factors, as well as in cellular response to bacterial invasion, enzymic deficiency, xenobiotics, and cytokines [126,127]. A few ROS examples include superoxide anion (O_2_), hydrogen peroxide (H_2_O_2_), and hydroxyl radical (HO•). Essential molecules such as DNA, proteins, and lipids are particularly vulnerable to ROS, whereas antioxidants protect these molecules from the negative effects of oxidation [128]. Oxidative stress is caused by an imbalance in antioxidant supply and oxidant component disposal (ROS) [129,130]. The antioxidative defense system is composed of antioxidant enzymes (catalase (CAT), glutathione-S-transferase (GST), glutathione peroxidase (GPx), and superoxide dismutase (SOD)) and non-enzymatic antioxidants (non-protein thiols (NPT)) (NPSH) [131,132,133]. The antioxidant activity is mediated by the reductive structure of the compound, which contains aromatic rings, phenolic compounds, and a high concentration of hydroxyl groups [134,135,136].

**Figure 1:**
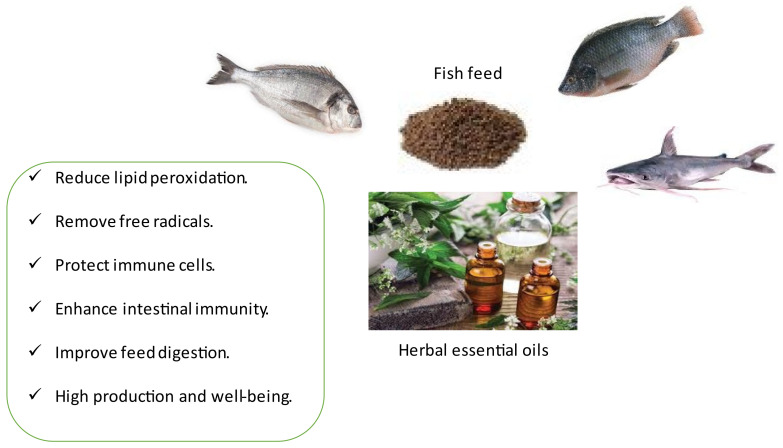
The roles of herbal essential oils on the performances of aquatic animals.

Several studies have indicated that natural antioxidants can improve the health status and performance of aquatic organisms [75,137,138,139,140,141,142,143,144,145,146] (Figure 1). In this context, an increase in antioxidant activity was found in koi carp fed diets with oregano EO (carvacrol and thymol) by Zhang et al. [99]. The same improvement in antioxidant activity with oregano EO (carvacrol and thymol) dietary supplements was determined in Nile tilapia [137,138], in rainbow trout [101], and in channel catfish [21,139]. The dietary incorporation of *A. triphylla* EO boosted the antioxidant status of silver catfish [140]. In addition, Sönmez et al. [120] declared a marked alteration in the antioxidant activity of rainbow trout juveniles fed diets containing sage, thyme, and mint EOs. Saccol et al. [80] reported that dietary supplementation of *L. alba* EO decreased lipid peroxidation and increased the tissue antioxidant response of silver catfish. Hsieh et al. [141] reported a strong antioxidant and anti-stress activity of rutin (bioflavonoid extracted from *Toona sinensis*) in white shrimp (*Litopenaeus vannamei*).

Moreover, research has indicated the role of EOs as an anesthetic in improving antioxidant activity. de Freitas Souza et al. [142] reported that anesthetics containing citral and linalool chemotypes of *L. alba* EO reduced lipid peroxidation while increasing the antioxidant activity in silver catfish. Similarly, Saccol et al. [143] found that rapid and extended sedation using Myrcia oil (*Myrcia sylvatica*) and turmeric oil (*Curcuma longa*) decreased lipid peroxidation and increased SOD, CAT, and GST in matrinxã (*Brycon amazonicus*). Barbas et al. [68] found that the use of *N. grandiflora* and *Spilanthes acmella* EOs as anesthetics boosted protection against muscular and gills oxidative damage of juvenile tambaqui (*Colossoma macropomum*). Silver catfish sedated with *A. triphylla* EO exhibited a lower level of lipid peroxides in the liver and higher CAT and GST activities [144]. Baldissera et al. [145] found that *M. alternifolia* EO helps to protect against oxidative damage in *R. quelen* infected with *Aeromonas hydrophila*.

## 5. Essential Oils as Immunostimulants

Herbal remedies include aromatherapy as a complementary medicine area, which has existed since ancient times, in which all or part of the plant/herb, extracts, or other herbal products are used via various administration methods (orally, topically, massaged, or inhaled) [146,147] (Figure 1). EOs extracted from plant sources possess distinctive antimicrobial, antioxidant, anti-inflammatory, anti-stress, appetite stimulators, analgesic, and aphrodisiac activities [73,148]. With the diversification of EO extraction methods (steam distillation, hydro diffusion, or pressure) and availability, applications and studies have increased [148,149] (Table 1). However, traditional extraction methods are worth close attention because these techniques take a long time, resulting in a reduction and degradation of specific volatile compounds [150]. Microwave-assisted, supercritical fluid, solvent extraction under pressure, and ultrasound-assisted extraction methods are more advanced to produce high-quality EOs with a low energy, cost, and less time [151].

Two categories of immune responses, namely the natural (innate) and the acquired (adaptive) immune responses, where immune stimulation is associated with the non-specific activation of both, enhance certain immune functions and thus the defense against various pathogens [152,153]. The innate response represents the first defensive action and a considerable part of the immunity system, which includes functions of monocytes, macrophages, basophil granulocytes, neutrophil, eosinophil mast cells, natural killer (NK) cells, and dendritic cells, and these functions involve phagocytosis, cytokine production, the release of inflammatory mediators, and antigen production [154]. Phagocytosis is a defensive line in fish that employs bactericidal and lysozyme activities as non-specific immune lines to tolerate pathogens [155,156]. The acquired response employs the production of antibodies/immunoglobulins (Ig), B cells (plasma cells), and T-cells (CD_4_^+^ T helper cells and CD_8_^+^ cytotoxic T cells) [148]. In fish, lymphocytes mediate cellular and humoral immune responses, and the primary lymph organs in fish are the kidney, spleen, thymus, and anterior [10]. 

Functional and nutritional supplements, as well as balanced diets, can stimulate immune responses in fish [157]. EOs have shown immunostimulant properties in several aquatic animals. In this regard, EOs from basil (*O. gratissimum*) and ginger (*Z. officinale*) in the diet boost the Nile tilapia’s immune system, increasing resistance to *S. agalactiae* and the phagocytic activity through increased thrombocytes, total leucocytes, lymphocytes, and neutrophils (THN) [115]. dos Santos et al. [158] reported that the inclusion of cinnamon oil (*Cinnamomum* sp.) in Nile tilapia diets subjected to acute hypoxic stress resulted in an increase of α1-, α2-globulins, and maintained the homeostasis of blood after hypoxic stress. In addition, the use of cinnamon powder elevated γ-globulin. Baba et al. [18] found an enhancement in the immune response of *O. mossambicus* fed *C. limon* peel EO and increasing resistance against the *Edwardsiella tarda* pathogen, highlighted by the enhancement in the nitro blue tetrazolium (NBT), total white blood cell (WBC), total protein (TP), lysozyme, and myeloperoxidase activities in the blood serum. Consistently, EOs originated from sweet orange peel boosted *O. mossambicus* defensive parameters, including activities of lysozyme and myeloperoxidase, and blood hematological and biochemical indices (i.e., serum total protein, hemoglobin, hematocrit levels, and erythrocyte) [17]. Sutili et al. [97] observed a significant improvement in the lysozyme activity in red drum fed a diet enhanced with *O. americanum* EO. In addition, the dietary addition of carvacrol improved some non-specific immune (lysozyme and myeloperoxidase activities) and serum biochemical statuses (total protein, globulin, triglyceride, and lower cholesterol) in rainbow trout [159]. Carvacrol and thymol stimulated the lymphocytes cell count in great sturgeon (*H. huso*) [121]. Moreover, *D. labrax* fed carvacrol exhibited remarkable resistance to the *Vibrio* pathogen and higher survival rates [160]. Dietary supplementation of carvacrol or thymol originated from *T. vulgaris* EO showed significant modulations in the lysozyme, the total amount of complement concentrations, and the catalase activity of rainbow trout [101]. Sheikhzadeh et al. [161] indicated that the inclusion of zaatar (*Zataria multiflora*) and blue gum (*Eucayptus globolus*) EOs in the diet is suggested to elevate the general wellbeing of common carp during thermal stress in terms of respiratory burst activity and blood hematological parameters (RBCs and haematocrit). Rattanachaikunsopon and Phumkhachorn [162] indicated that cinnamon oil, which consists of 90.24% cinnamaldehyde, 2.42% limonene, 2.03% cinnamyl acetate, 1.16% linalool, and 0.87% α-terpineol, had a protective potent effect on experimental *Streptococcus iniae* infection in Nile tilapia. The incorporation of *Z. multiflora* EO in common carp diets enhanced immunity during low temperatures.

## 6. Concluding Remarks and Future Outlook

Herbal EOs provide enormous beneficial effects in aquaculture by improving appetite, microbial balance, immune responses, antioxidative capacity, and disease resistance of aquatic animals. At the same time, EOs provide growth-promoting and feed utilization effects. A comprehensive review indicates that the primary determinants of EO efficacy in aquatic animals are the oil’s source, concentration, and duration of administration. This review article clearly illustrates that herbal EOs have beneficial effects on aquatic animals’ performances, and can feasibly replace antibiotics and chemotherapies for clean, healthy, and sustainable aquaculture.

The gut microbiome, metabolomics, and proteomic tools should be taken into consideration to determine the potential impacts of EOs and their mechanisms on the immune system, gut microbiota, and growth performance. Hence, further studies on fish transcriptomic profiles are also required to determine and quantify the effects of botanical EO concentrations on adaptive immune response, antioxidative status, and disease resilience. Furthermore, further research plans are needed in this direction, coupled with comprehensive studies using advanced methods to characterize the gut microbiota of targeted fish species. Additional research is also required to investigate the possibility of combining EOs with other feed additives (e.g., probiotics and prebiotics) and comparing their effects to antibiotics.

## Figures and Tables

**Table 1:** Herbal essential oils and their impacts on the physical performances and physiological responses of aquatic animals.

Aquatic Species	Essential Oil	Dose and Duration	Influence	Reference
Common carp (*Cyprinus carpio*)	*Zataria multiflora*	30–120 ppm/kg diet for 22 days	- Antibody titer, WBCs, and bactericidal activity (↑) - Resistance against heat stress (↑)	[163]
Silver catfish (*Rhamdia quelen*)	Lippia alba	10 μL/L for 7 h	- LPO, CAT, SOD, and GST in the liver, gills, and brain (↓) - Resistance against transport stress (↑)	[164]
Rainbow trout (*Oncorhynchus mykiss*)	Black cumin seed oil	1, 2, and 3 for 14 days	- Lysozyme, total protein, antiprotease, total serum IgM, and bactericidal activity (↑)	[165]
Rainbow trout (*Oncorhynchus mykiss*)	Carvacrol and thymol	1 g/kg for 8 weeks	- Feed efficiency, lysozyme, total complement concentrations, and CAT (↑) - Growth performance ( 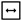 ) - MDA, total anaerobe counts, and *Lactobacillus* loads (↓)	[101]
Silver catfish (*Rhamdia quelen*)	Lippia alba	0.25, 0.5, 1.0, or 2.0 mL/kg diet for 60 days	- Growth performance and blood indices ( 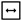 ) - SOD, CAT, GPx, and GST (brain, gills, liver, kidney, and muscle) (↑)	[80]
Red drum (*Sciaenops ocellatus*)	Lime basil	0, 0.25, 0.5, 1.0, and 2.0 g/kg diet for 7 weeks	- Growth performance and intestinal microbial community ( 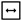 ) - Intraperitoneal fat deposition and stomach lysozyme activity (↑)	[97]
Nile tilapia (*Oreochromis niloticus*)	Limonene and thymol	0, 200, 400, and 600 mg/kg for 63 days	- Growth performance, IGF-I, MUC, PEPT1, LPL, ALP and CAT (↑)	[114]
Common carp (*Cyprinus carpio*)	Blue gum	30, 60, and 120 µL/L or mg/kg feed for 8 days	- Antibody titers and total white blood cells (↑) - Resistance against low water temperature (↑)	[166]
Nile tilapia (*Oreochromis niloticus*)	Pepper rosemary and peppermint	20–40 mg/L (3 baths for 10 min each)	- The monogenean parasite prevalence (↓) - RBC and thrombocytes with *L. sidoides* (↓) - Glucose concentration and neutrophil count with *L. sidoides* (↑)	[76]
Rainbow trout (*Oncorhynchus mykiss*)	Carvacrol	0, 1, 3, or 5 g/kg for 60 days	- Lysozyme and myeloperoxidase activities (↑) - The serum total protein and globulin (↑) - Glucose and triglyceride (↓)	[159]
Common carp (*Cyprinus carpio* L.)	Oregano	0, 5, 10, 15, and 20 g/kg diet for 2 months	- Growth indices and feed utilization (↑) - Total protein, albumin, and globulin, AST, ALP, ALT, and renal markers (creatinine and urea) ( 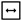 ) - Intestinal morphometric measurements (↑)	[108]
Nile tilapia (*Oreochromis niloticus*)	Peppermint and tea tree	100 and 250 mg/kg for 60 days	- The haematological and biochemical parameters ( 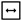 ) - The complement system (↑) - Intestinal morphology (↑)	[167]
Rainbow trout (*Oncorhynchus mykiss*)	1,8-cineole, carvacrol or pulegone	0.5, 1, and 1.5% for 60 days	- Growth indices and feed utilization (↑) - Liver or kidney histological alterations ( 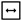 )	[168]
Rainbow trout (*Oncorhynchus mykiss*)	Oregano	6 and 10 g/kg diet	- TAC, SOD, CAT, and GPX (↑) - MDA, ALT, AST, and LHD ( 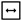 ) - Resistance against diazinon toxicity (↑)	[169]
Yellowtail Tetra (*Astyanax altiparanae*)	Oregano	0.0, 0.5, 1.0, 1.5, 2, and 2.5 g/kg for 90 days	- Growth indices and feed utilization (↑)	[170]
Nile tilapia (*Oreochromis niloticus*)	Oregano	0.0, 1.0, and 2 mL/kg for 10 weeks	- Growth indices and feed utilization (↑) - SOD, GR, and NO (↑) - Resistance against stocking density (↑)	[138]
Nile tilapia (*Oreochromis niloticus*)	Cinnamaldehyde and thymol	1 and 2 mL/kg diet for 75 days	- Growth indices and feed utilization (↑) - GR, lysozyme activity, IgM, IgG levels, and CAT (↑) - MDA (↓)	[113]
Great sturgeon (*Huso huso* Linnaeus, 1758)	Thymol–carvacrol	0, 1.0, 2.0, and 3.0 g/kg for 60 days	- Growth indices and feed utilization (↑) - Haematological indices ( 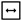 )	[121]
Nile tilapia (*Oreochromis niloticus*)	Menthol	0.25% for 30 days	- Growth indices and feed utilization (↑) - Antioxidative capacity (↑) - Immune response, anti-inflammatory, and proinflammatory related genes (↑) - Resistance against chlorpyrifos toxicity (↑)	[171]

(↑): significantly increases; (↓): significantly decreased; (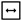): no significant change; WBCs: white blood cells; LPO: lipoperoxidation; CAT: catalase; SOD: superoxide dismutase; GST: glutathione-S-transferase; IgM: immunoglobulin; MDA: malondialdehyde; IGF-I: insulin growth factor I; MUC: mucin-like protein; PEPT1: oligo-peptide transporter I; LPL: lipoprotein lipase; ALP: alkaline phosphatase; RBC: red blood cells; TAC: total antioxidant capacity; LHD: lactate dehydrogenase; ALT: alanine aminotransferase; AST: aspartate aminotransferase; GR: glutathione reductase; NO: nitric oxide.

## Data Availability

Not applicable.

## Supplementary Materials

The following supporting information can be downloaded at: https://www.mdpi.com/article/10.3390/ani12070823/s1. Table S1: Linalool in the essential oil constituents of its natural sources (>10%). Table S2: Myrcene in the essential oil constituents of its natural sources (>10%). Table S3: Eucalyptol (1,8-cineole) in the essential oil constituents of its natural sources (>10%). Table S4: β-caryophyllene in the essential oil constituents of its natural sources (>10%).

## References

[1] Tacon A.G.J. (2020). Trends in global aquaculture and aquafeed production: 2000–2017. Rev. Fish. Sci. Aquac..

[2] Dawood M.A.O., Noreldin A.E., Sewilam H. (2021). Long term salinity disrupts the hepatic function, intestinal health, and gills antioxidative status in nile tilapia stressed with hypoxia. Ecotoxicol. Environ. Saf..

[3] Dawood M.A.O. (2021). Nutritional immunity of fish intestines: Important insights for sustainable aquaculture. Rev. Aquac..

[4] Garlock T., Asche F., Anderson J., Bjørndal T., Kumar G., Lorenzen K., Ropicki A., Smith M.D., Tveterås R. (2020). A global blue revolution: Aquaculture growth across regions, species, and countries. Rev. Fish. Sci. Aquac..

[5] Dawood M.A.O., Koshio S., Esteban M.Á. (2018). Beneficial roles of feed additives as immunostimulants in aquaculture: A review. Rev. Aquac..

[6] Yukgehnaish K., Kumar P., Sivachandran P., Marimuthu K., Arshad A., Paray B.A., Arockiaraj J. (2020). Gut microbiota metagenomics in aquaculture: Factors influencing gut microbiome and its physiological role in fish. Rev. Aquac..

[7] Mohammadi G., Hafezieh M., Karimi A.A., Azra M.N., Van Doan H., Tapingkae W., Abdelrahman H.A., Dawood M.A.O. (2022). The synergistic effects of plant polysaccharide and *Pediococcus acidilactici* as a synbiotic additive on growth, antioxidant status, immune response, and resistance of Nile tilapia (*Oreochromis niloticus*) against *Aeromonas hydrophila*. Fish Shellfish Immunol..

[8] Shourbela R.M., El-Hawarry W.N., Elfadadny M.R., Dawood M.A.O. (2021). Oregano essential oil enhanced the growth performance, immunity, and antioxidative status of Nile tilapia (*Oreochromis niloticus*) reared under intensive systems. Aquaculture.

[9] Elumalai P., Kurian A., Lakshmi S., Faggio C., Esteban M.A., Ringø E. (2020). Herbal immunomodulators in aquaculture. Rev. Fish. Sci. Aquac..

[10] Vaseeharan B., Thaya R. (2014). Medicinal plant derivatives as immunostimulants: An alternative to chemotherapeutics and antibiotics in aquaculture. Aquac. Int..

[11] Aydın B., Barbas L.A.L. (2020). Sedative and anesthetic properties of essential oils and their active compounds in fish: A review. Aquaculture.

[12] Zeng Z., Zhang S., Wang H., Piao X. (2015). Essential oil and aromatic plants as feed additives in non-ruminant nutrition: A review. J. Anim. Sci. Biotechnol..

[13] Abdelkhalek N.K., Risha E., El-Adl M.A., Salama M.F., Dawood M.A.O. (2020). Antibacterial and antioxidant activity of clove oil against *Streptococcus iniae* infection in Nile tilapia (*Oreochromis niloticus*) and its effect on hepatic hepcidin expression. Fish Shellfish Immunol..

[14] Alagawany M., Farag M.R., Salah A.S., Mahmoud M.A. (2020). The role of oregano herb and its derivatives as immunomodulators in fish. Rev. Aquac..

[15] Ghafarifarsani H., Kachuei R., Imani A. (2021). Dietary supplementation of garden thyme essential oil ameliorated the deteriorative effects of aflatoxin B_1_ on growth performance and intestinal inflammatory status of rainbow trout (*Oncorhynchus mykiss*). Aquaculture.

[16] Dawood M.A.O., El Basuini M.F., Zaineldin A.I., Yilmaz S., Hasan M.T., Ahmadifar E., El Asely A.M., Abdel-Latif H.M.R., Alagawany M., Abu-Elala N.M. (2021). Antiparasitic and antibacterial functionality of essential oils: An alternative approach for sustainable aquaculture. Pathogens.

[17] Acar U., Kesbiç O.S., Yilmaz S., Gültepe N., Türker A. (2015). Evaluation of the effects of essential oil extracted from sweet orange peel (*Citrus sinensis*) on growth rate of tilapia (*Oreochromis mossambicus*) and possible disease resistance against *Streptococcus iniae*. Aquaculture.

[18] Baba E., Acar Ü., Öntaş C., Kesbiç O.S., Yılmaz S. (2016). Evaluation of citrus limon peels essential oil on growth performance, immune response of Mozambique tilapia *Oreochromis mossambicus* challenged with *Edwardsiella tarda*. Aquaculture.

[19] Ngugi C.C., Oyoo-Okoth E., Muchiri M. (2017). Effects of dietary levels of essential oil (eo) extract from bitter lemon (*Citrus limon*) fruit peels on growth, biochemical, haemato-immunological parameters and disease resistance in juvenile *Labeo victorianus* fingerlings challenged with *Aeromonas hydrophila*. Aquac. Res..

[20] Abdel-Latif H.M.R., Abdel-Tawwab M., Khafaga A.F., Dawood M.A.O. (2020). Dietary origanum essential oil improved antioxidative status, immune-related genes, and resistance of common carp (*Cyprinus carpio* L.) to *Aeromonas hydrophila* infection. Fish Shellfish Immunol..

[21] Zheng Z.L., Tan J.Y.W., Liu H.Y., Zhou X.H., Xiang X., Wang K.Y. (2009). Evaluation of oregano essential oil (*Origanum heracleoticum* L.) on growth, antioxidant effect and resistance against *Aeromonas hydrophila* in channel catfish (*Ictalurus punctatus*). Aquaculture.

[22] Diler O., Gormez O., Diler I., Metin S. (2017). Effect of oregano (*Origanum onites* L.) essential oil on growth, lysozyme and antioxidant activity and resistance against *Lactococcus garvieae* in rainbow trout, *Oncorhynchus mykiss* (walbaum). Aquac. Nutr..

[23] Anastasiou T.I., Mandalakis M., Krigas N., Vézignol T., Lazari D., Katharios P., Dailianis T., Antonopoulou E. (2020). Comparative evaluation of essential oils from medicinal-aromatic plants of greece: Chemical composition, antioxidant cpacity and antimicrobial activity against bacterial fish pathogens. Molecules.

[24] Ahmadifar E., Yousefi M., Karimi M., Fadaei Raieni R., Dadar M., Yilmaz S., Dawood M.A.O., Abdel-Latif H.M.R. (2021). Benefits of dietary polyphenols and polyphenol-rich additives to aquatic animal health: An overview. Rev. Fish. Sci. Aquac..

[25] Bakkali F., Averbeck S., Averbeck D., Idaomar M. (2008). Biological effects of essential oils—A review. Food Chem. Toxicol..

[26] Carson C.F., Hammer K.A. (2010). Chemistry and Bioactivity of Essential Oils.

[27] Hüsnü K., Baśer C., Demirci F. (2007). Chemistry of Essential Oils.

[28] Bilia A.R., Guccione C., Isacchi B., Righeschi C., Firenzuoli F., Bergonzi M.C. (2014). Essential oils loaded in nanosystems: A developing strategy for a successful therapeutic approach. Evid.-Based Complementary Altern. Med..

[29] De FreitasSouza C., Baldissera M.D., Baldisserotto B., Heinzmann B.M., Martos-Sitcha J.A., Mancera J.M. (2019). Essential oils as stress-reducing agents for fish aquaculture: A review. Front. Physiol..

[30] Góra J., Lis A., Kula J., Staniszewska M., Wołoszyn A. (2002). Chemical composition variability of essential oils in the ontogenesis of some plants. Flavour Fragr. J..

[31] Azmir J., Zaidul I.S.M., Rahman M.M., Sharif K.M., Mohamed A., Sahena F., Jahurul M.H.A., Ghafoor K., Norulaini N.A.N., Omar A.K.M. (2013). Techniques for extraction of bioactive compounds from plant materials: A review. J. Food Eng..

[32] Tongnuanchan P., Benjakul S. (2014). Essential oils: Extraction, bioactivities, and their uses for food preservation. J. Food Sci..

[33] Manuale D.L., Betti C., Marchi A.J., Yori J.C., Romeo E. (2010). Synthesis of liquid menthol by hydrogenation of dementholized peppermint oil over ni catalysts. Quim. Nova.

[34] Patel T., Ishiuji Y., Yosipovitch G. (2007). Menthol: A refreshing look at this ancient compound. J. Am. Acad. Dermatol..

[35] Kamatou G.P.P., Vermaak I., Viljoen A.M., Lawrence B.M. (2013). Menthol: A simple monoterpene with remarkable biological properties. Phytochemistry.

[36] Hoseini S.M., Taheri Mirghaed A., Yousefi M. (2019). Application of herbal anaesthetics in aquaculture. Rev. Aquac..

[37] Kalemba D., Synowiec A. (2020). Agrobiological interactions of essential oils of two menthol mints: *Mentha piperita* and *Mentha arvensis*. Molecules.

[38] Tisserand R., Young R. (2014). Essential Oil Safety: A Guide for Health Care Professionals.

[39] Etzold B., Jess A., Nobis M. (2009). Epimerisation of menthol stereoisomers: Kinetic studies of the heterogeneously catalysed menthol production. Catal. Today.

[40] Elsharif S.A., Banerjee A., Buettner A. (2015). Structure-odor relationships of linalool, linalyl acetate and their corresponding oxygenated derivatives. Front. Chem..

[41] Stashenko E.E., Martínez J.R. (2008). Sampling flower scent for chromatographic analysis. J. Sep. Sci..

[42] Behr A., Johnen L. (2009). Myrcene as a natural base chemical in sustainable chemistry: A critical review. ChemSusChem.

[43] Giese M.W., Lewis M.A., Giese L., Smith K.M. (2015). Method for the analysis of cannabinoids and terpenes in cannabis. J. AOAC Int..

[44] Hazekamp A., Tejkalová K., Papadimitriou S. (2016). Cannabis: From cultivar to chemovar ii—A metabolomics approach to cannabis classification. Cannabis Cannabinoid Res..

[45] Russo E.B., Marcu J., Kendall D., Alexander S.P.H. (2017). Chapter three-cannabis pharmacology: The usual suspects and a few promising leads. Advances in Pharmacology.

[46] Aprotosoaie A.C., Luca V.S., Trifan A., Miron A. (2018). Antigenotoxic Potential of Some Dietary Non-Phenolic Phytochemicals.

[47] Flamini G., Attaur R. (2012). Chapter 13-natural herbicides as a safer and more environmentally friendly approach to weed control: A review of the literature since 2000. Studies in Natural Products Chemistry.

[48] Barbosa L., Filomeno C., Teixeira R. (2016). Chemical variability and biological activities of *Eucalyptus* spp. Assential oils. Molecules.

[49] Barnes J., Barnes J., Anderson L., Phillipson D. (2007). Herbal Medicines.

[50] Tan M., Zhou L., Huang Y., Wang Y., Hao X., Wang J. (2008). Antimicrobial activity of globulol isolated from the fruits of *Eucalyptus globulus* labill. Nat. Prod. Res..

[51] Morais S.M., Cossolosso D.S., Silva A.A.S., de Moraes Filho M.O., Teixeira M.J., Campello C.C., Bonilla O.H., de Paula V.F., Vila-Nova N.S. (2019). Essential oils from croton species: Chemical composition, in vitro and in silico antileishmanial evaluation, antioxidant and cytotoxicity activities. J. Braz. Chem. Soc..

[52] Mendes S., Nunes D., Marques M., Tardivo R., Cechinel Filho V., Siminonatto E., Wisniewski A. (2008). Essential oil of baccharis semiserrata, a source of spathulenol. Publ. UEPG-Cienc. Exatas E Da Terra Agrar. E Eng..

[53] Apel M.A., Lima M.E.L., Sobral M., Young M.C.M., Cordeiro I., Schapoval E.E.S., Henriques A.T., Moreno P.R.H. (2010). Anti-inflammatory activity of essential oil from leaves of *Myrciaria tenella* and *Calycorectes sellowianus*. Pharm. Biol..

[54] Kula J., Majda T., Stoyanova A., Georgiev E. (2007). Chemical composition of *Origanum vulgare* L. Essential oil from Bulgaria. J. Essent. Oil-Bear. Plants.

[55] Hillig K.W. (2004). A chemotaxonomic analysis of terpenoid variation in cannabis. Biochem. Syst. Ecol..

[56] Kamal B.S., Kamal F., Lantela D.E. (2018). Cannabis and the anxiety of fragmentation—A systems approach for finding an anxiolytic cannabis chemotype. Front. Neurosci..

[57] Yang Q., Wu J., Luo Y., Huang N., Zhen N., Zhou Y., Sun F., Li Z., Pan Q., Li Y. (2016). (−)-guaiol regulates rad51 stability via autophagy to induce cell apoptosis in non-small cell lung cancer. Oncotarget.

[58] Farag R.S., Shalaby A.S., El-Baroty G.A., Ibrahim N.A., Ali M.A., Hassan E.M. (2004). Chemical and biological evaluation of the essential oils of different melaleuca species. Phytother. Res..

[59] Clarke S. (2008). Families of Compounds that Occur in Essential Oils.

[60] Jafri H., Ansari F.A., Ahmad I. (2018). Prospects of Essential Oils in Controlling Pathogenic Biofilm.

[61] Can Baser K. (2008). Biological and pharmacological activities of carvacrol and carvacrol bearing essential oils. Curr. Pharm. Des..

[62] Noma Y., Asakawa Y. (2010). Biotransformation of Monoterpenoids.

[63] Benelli G., Canale A., Flamini G., Cioni P.L., Demi F., Ceccarini L., Macchia M., Conti B. (2013). Biotoxicity of *Melaleuca alternifolia* (myrtaceae) essential oil against the mediterranean fruit fly, *Ceratitis capitata* (diptera: Tephritidae), and its parasitoid *Psyttalia concolor* (hymenoptera: Braconidae). Ind. Crops Prod..

[64] Gómez-Rincón C., Langa E., Murillo P., Valero M.S., Berzosa C., López V. (2014). Activity of tea tree (*Melaleuca alternifolia*) essential oil against l3 larvae of *Anisakis simplex*. BioMed Res. Int..

[65] Shapira S., Pleban S., Kazanov D., Tirosh P., Arber N. (2016). Terpinen-4-ol: A novel and promising therapeutic agent for human gastrointestinal cancers. PLoS ONE.

[66] Yu D., Wang J., Shao X., Xu F., Wang H. (2015). Antifungal modes of action of tea tree oil and its two characteristic components against (*Botrytis cinerea*). J. Appl. Microbiol..

[67] Hart P.H., Brand C., Carson C.F., Riley T.V., Prager R.H., Finlay-Jones J.J. (2000). Terpinen-4-ol, the main component of the essential oil of *Melaleuca alternifolia* (tea tree oil), suppresses inflammatory mediator production by activated human monocytes. Inflamm. Res..

[68] Barbas L.A.L., Maltez L.C., Stringhetta G.R., Garcia L.d.O., Monserrat J.M., da Silva D.T., Heinzmann B.M., Sampaio L.A. (2017). Properties of two plant extractives as anaesthetics and antioxidants for juvenile tambaqui *Colossoma macropomum*. Aquaculture.

[69] Garlet Q.I., Pires L.d.C., Milanesi L.H., Marafiga J.R., Baldisserotto B., Mello C.F., Heinzmann B.M. (2017). (+)-dehydrofukinone modulates membrane potential and delays seizure onset by gabaa receptor-mediated mechanism in mice. Toxicol. Appl. Pharmacol..

[70] Hosseini M., Jamshidi A., Raeisi M., Azizzadeh M. (2019). The antibacterial andaantioxidant effects of clove (*Syzygium aromaticum*) and lemon verbena (*Aloysia citriodora*) essential oils. J. Hum. Environ. Health Promot..

[71] Galvez C.E., Jimenez C.M., Gomez A.d.l.A., Lizarraga E.F., Sampietro D.A. (2020). Chemical composition and antifungal activity of essential oils from *Senecio nutans*, *Senecio viridis*, *Tagetes terniflora* and *Aloysia gratissima* against toxigenic *Aspergillus* and *Fusarium* species. Nat. Prod. Res..

[72] Verni M.C., Garay J.A., Mendoza L., Bardón A., Borkosky S., Arena M.E., Cartagena E. (2020). Lipophilic 9,10-dehydrofukinone action on pathogenic and non-pathogenic bacterial biofilms. Why is this main volatile metabolite in senecio?. Chem. Biodivers..

[73] Citarasu T. (2010). Herbal biomedicines: A new opportunity for aquaculture industry. Aquac. Int..

[74] El Basuini M.F., Teiba I.I., Zaki M.A.A., Alabssawy A.N., El-Hais A.M., Gabr A.A., Dawood M.A.O., Zaineldin A.I., Mzengereza K., Shadrack R.S. (2020). Assessing the effectiveness of COQ10 dietary supplementation on growth performance, digestive enzymes, blood health, immune response, and oxidative-related genes expression of Nile tilapia (*Oreochromis niloticus*). Fish Shellfish Immunol..

[75] Sutili F.J., Gatlin D.M., Heinzmann B.M., Baldisserotto B. (2018). Plant essential oils as fish diet additives: Benefits on fish health and stability in feed. Rev. Aquac..

[76] de Oliveira Hashimoto G.S., Neto F.M., Ruiz M.L., Acchile M., Chagas E.C., Chaves F.C.M., Martins M.L. (2016). Essential oils of lippia sidoides and *Mentha piperita* against monogenean parasites and their influence on the hematology of Nile tilapia. Aquaculture.

[77] Harikrishnan R., Balasundaram C., Heo M.-S. (2011). Impact of plant products on innate and adaptive immune system of cultured finfish and shellfish. Aquaculture.

[78] Penino N.C., Santos G.D.O., Rodrigues M.F., Bastos H.B.D.A., Winter G.H.Z., Bustamante-Filho I.C., Pimentel A.M., Gregory R.M., Mattos R.C. (2015). Effect of intramuscular injection of butafosfan and cobalamin on the quality of fresh and cooled stallion semen. Semin. Cienc. Agrar..

[79] Reverter M., Bontemps N., Lecchini D., Banaigs B., Sasal P. (2014). Use of plant extracts in fish aquaculture as an alternative to chemotherapy: Current status and future perspectives. Aquaculture.

[80] Saccol E.M.H., Uczay J., Pês T.S., Finamor I.A., Ourique G.M., Riffel A.P.K., Schmidt D., Caron B.O., Heinzmann B.M., Llesuy S.F. (2013). Addition of *Lippia alba* (mill) n. E. Brown essential oil to the diet of the silver catfish: An analysis of growth, metabolic and blood parameters and the antioxidant response. Aquaculture.

[81] Sutili F.J., de Lima Silva L., Gressler L.T., Gressler L.T., Battisti E.K., Heinzmann B.M., de Vargas A.C., Baldisserotto B. (2015). Plant essential oils against *Aeromonas hydrophila*: *In vitro* activity and their use in experimentally infected fish. J. Appl. Microbiol..

[82] Domadia P., Swarup S., Bhunia A., Sivaraman J., Dasgupta D. (2007). Inhibition of bacterial cell division protein ftsz by cinnamaldehyde. Biochem. Pharmacol..

[83] Nazzaro F., Fratianni F., De Martino L., Coppola R., De Feo V. (2013). Effect of essential oils on pathogenic bacteria. Pharmaceuticals.

[84] Silva N.C.C., Fernandes Júnior A. (2010). Biological properties of medicinal plants: A review of their antimicrobial activity. J. Venom. Anim. Toxins Incl. Trop. Dis..

[85] Togashi N., Inoue Y., Hamashima H., Takano A. (2008). Effects of two terpene alcohols on the antibacterial activity and the mode of action of farnesol against *Staphylococcus aureus*. Molecules.

[86] Laparra J.M., Sanz Y. (2010). Interactions of gut microbiota with functional food components and nutraceuticals. Pharmacol. Res..

[87] Gómez G.D., Balcázar J.L. (2008). A review on the interactions between gut microbiota and innate immunity of fish: Table 1. FEMS Immunol. Med. Microbiol..

[88] Pérez T., Balcázar J.L., Ruiz-Zarzuela I., Halaihel N., Vendrell D., de Blas I., Múzquiz J.L. (2010). Host–microbiota interactions within the fish intestinal ecosystem. Mucosal Immunol..

[89] Teiba I., Okunishi S., Yoshikawa T., Ikenaga M., Fouad El Basuini M., Mae S Santander-De Leon S., Maeda H. (2020). Use of purple non-sulfur photosynthetic bacteria (*Rhodobacter sphaeroides*) in promoting ciliated protozoa growth. Biocontrol Sci..

[90] Teiba I., Yoshikawa T., Okunishi S., Ikenaga M., Basuini M.E., Maeda H. (2020). Diversity of the photosynthetic bacterial communities in highly eutrophicated Yamagawa bay sediments. Biocontrol Sci..

[91] Ellis A.E. (2001). Innate host defense mechanisms of fish against viruses and bacteria. Dev. Comp. Immunol..

[92] Si W., Gong J., Tsao R., Zhou T., Yu H., Poppe C., Johnson R., Du Z. (2006). Antimicrobial activity of essential oils and structurally related synthetic food additives towards selected pathogenic and beneficial gut bacteria. J. Appl. Microbiol..

[93] Thapa D., Losa R., Zweifel B., John Wallace R. (2012). Sensitivity of pathogenic and commensal bacteria from the human colon to essential oils. Microbiology.

[94] Bento M.H.L., Ouwehand A.C., Tiihonen K., Lahtinen S., Nurminen P., Saarinen M.T., Schulze H., Mygind T., Fischer J. (2013). Essential oils and their use in animal feeds for monogastric animals—Effects on feed quality, gut microbiota, growth performance and food safety: A review. Vet. Med..

[95] Chakraborty S.B., Horn P., Hancz C. (2014). Application of Phytochemicals as Growth-Promoters and Endocrine Modulators in Fish Culture.

[96] Franz C., Baser K.H.C., Windisch W. (2010). Essential oils and aromatic plants in animal feeding–A European perspective. A review. Flavour Fragr. J..

[97] Sutili F.J., Velasquez A., Pinheiro C.G., Heinzmann B.M., Gatlin D.M., Baldisserotto B. (2016). Evaluation of ocimum americanum essential oil as an additive in red drum (*Sciaenops ocellatus*) diets. Fish Shellfish Immunol..

[98] Navarrete P., Toledo I., Mardones P., Opazo R., Espejo R., Romero J. (2010). Effect of *Thymus vulgaris* essential oil on intestinal bacterial microbiota of rainbow trout, *Oncorhynchus mykiss* (walbaum) and bacterial isolates. Aquac. Res..

[99] Zhang R., Wang X.W., Liu L.L., Cao Y.C., Zhu H. (2020). Dietary oregano essential oil improved the immune response, activity of digestive enzymes, and intestinal microbiota of the koi carp, *Cyprinus carpio*. Aquaculture.

[100] Al-Sagheer A.A., Mahmoud H.K., Reda F.M., Mahgoub S.A., Ayyat M.S. (2018). Supplementation of diets for *Oreochromis niloticus* with essential oil extracts from lemongrass (*Cymbopogon citratus*) and geranium (*Pelargonium graveolens*) and effects on growth, intestinal microbiota, antioxidant and immune activities. Aquac. Nutr..

[101] Giannenas I., Triantafillou E., Stavrakakis S., Margaroni M., Mavridis S., Steiner T., Karagouni E. (2012). Assessment of dietary supplementation with carvacrol or thymol containing feed additives on performance, intestinal microbiota and antioxidant status of rainbow trout (*Oncorhynchus mykiss*). Aquaculture.

[102] Ran C., Hu J., Liu W., Liu Z., He S., Dan B.C.T., Diem N.N., Ooi E.L., Zhou Z. (2016). Thymol and carvacrol affect hybrid tilapia through the combination of direct stimulation and an intestinal microbiota-mediated effect: Insights from a germ-free zebrafish model. J. Nutr..

[103] Hernández F., Madrid J., García V., Orengo J., Megías M.D. (2004). Influence of two plant extracts on broilers performance, digestibility, and digestive organ size. Poult. Sci..

[104] Michiels J., Missotten J., van Hoorick A., Ovyn A., Fremaut D., de Smet S., Dierick N. (2010). Effects of dose and formulation of carvacrol and thymol on bacteria and some functional traits of the gut in piglets after weaning. Arch. Anim. Nutr..

[105] Venketramalingam K., Christopher J.G., Citarasu T. (2007). *Zingiber officinalis* an herbal appetizer in the tiger shrimp *Penaeus monodon* (fabricius) larviculture. Aquac. Nutr..

[106] Adeshina I., Jenyo-Oni A., Emikpe B.O., Ajani E.K., Abdel-Tawwab M. (2019). Stimulatory effect of dietary clove, *Eugenia caryophyllata*, bud extract on growth performance, nutrient utilization, antioxidant capacity, and tolerance of African catfish, *Clarias gariepinus* (b.), to *Aeromonas hydrophila* infection. J. World Aquac. Soc..

[107] Huerta-Aguirre G., Paredes-Ramos K.M., Becerra-Amezcua M.P., Hernández-Calderas I., Matadamas-Guzman M., Guzmán-García X. (2019). Histopathological Analysis of the Intestine from Mugil cephalus on Environment Reference Sites.

[108] Abdel-Latif H.M.R., Abdel-Tawwab M., Khafaga A.F., Dawood M.A.O. (2020). Dietary oregano essential oil improved the growth performance via enhancing the intestinal morphometry and hepato-renal functions of common carp (*Cyprinus carpio* L.) fingerlings. Aquaculture.

[109] Abd El-Naby A.S., Al-Sagheer A.A., Negm S.S., Naiel M.A.E. (2020). Dietary combination of chitosan nanoparticle and thymol affects feed utilization, digestive enzymes, antioxidant status, and intestinal morphology of *Oreochromis niloticus*. Aquaculture.

[110] Ferreira P.M.F., Caldas D.W., Salaro A.L., Sartori S.S.R., Oliveira J.M., Cardoso A.J.S., Zuanon J.A.S. (2016). Intestinal and liver morphometry of the yellow tail tetra (*Astyanax altiparanae*) fed with oregano oil. An. Da Acad. Bras. De Cienc..

[111] de Oliveira S.T.L., Soares R.A.N., de Negreiros Sousa S.M., Fernandes A.W.C., Gouveia G.V., da Costa M.M. (2020). Natural products as functional food ingredients for Nile tilapia challenged with *Aeromonas hydrophila*. Aquac. Int..

[112] Rattanachaikunsopon P., Phumkhachorn P. (2010). Assessment of synergistic efficacy of carvacrol and cymene against *Edwardsiella tarda* in vitro and in tilapia (*Oreochromis niloticus*). Afr. J. Microbiol. Res..

[113] Amer S.A., Metwally A.E., Ahmed S.A.A. (2018). The influence of dietary supplementation of cinnamaldehyde and thymol on the growth performance, immunity and antioxidant status of monosex Nile tilapia fingerlings (*Oreochromis niloticus*). Egypt. J. Aquat. Res..

[114] Aanyu M., Betancor M.B., Monroig O. (2018). Effects of dietary limonene and thymol on the growth and nutritional physiology of Nile tilapia (*Oreochromis niloticus*). Aquaculture.

[115] Brum A., Pereira S.A., Owatari M.S., Chagas E.C., Chaves F.C.M., Mouriño J.L.P., Martins M.L. (2017). Effect of dietary essential oils of clove basil and ginger on Nile tilapia (*Oreochromis niloticus*) following challenge with *Streptococcus agalactiae*. Aquaculture.

[116] Abo-State H.A., El-Monairy M.M., Hammouda Y.A., Elgendy M.Y. (2017). Effect of a phytogenic feed additive on the growth performance and susceptibility of *Oreochromis niloticus* to *Aeromonas hydrophila*. J. Fish. Aquat. Sci..

[117] Hassaan M.S., Soltan M.A. (2016). Evaluation of essential oil of fennel and garlic separately or combined with *Bacillus licheniformis* on the growth, feeding behaviour, hemato-biochemical indices of *Oreochromis niloticus* (L.) fry. J. Aquac. Res. Dev..

[118] Zeppenfeld C.C., Hernández D.R., Santinón J.J., Heinzmann B.M., da Cunha M.A., Schmidt D., Baldisserotto B. (2016). Essential oil of *Aloysia triphylla* as feed additive promotes growth of silver catfish (*Rhamdia quelen*). Aquac. Nutr..

[119] Midhun S.J., Arun D., Edatt L., Sruthi M.V., Thushara V.V., Oommen O.V., Sameer Kumar V.B., Divya L. (2016). Modulation of digestive enzymes, gh, igf-1 and igf-2 genes in the teleost, tilapia (*Oreochromis mossambicus*) by dietary curcumin. Aquac. Int..

[120] Sönmez A.Y., Bilen S., Alak G., Hisar O., Yanık T., Biswas G. (2015). Growth performance and antioxidant enzyme activities in rainbow trout (*Oncorhynchus mykiss*) juveniles fed diets supplemented with sage, mint and thyme oils. Fish Physiol. Biochem..

[121] Ahmadifar E., Razeghi Mansour M., Keramat Amirkolaie A., Fadaii Rayeni M. (2014). Growth efficiency, survival and haematological changes in great sturgeon (*Huso huso* linnaeus, 1758) juveniles fed diets supplemented with different levels of thymol–carvacrol. Anim. Feed Sci. Technol..

[122] Ferreira P.d.M.F., Nascimento L.d.S., Dias D.C., Moreira D.M.d.V., Salaro A.L., de Freitas M.B.D., Carneiro A.P.S., Zuanon J.A.S. (2014). Essential oregano oil as a growth promoter for the yellowtail tetra, *Astyanax altiparanae*. J. World Aquac. Soc..

[123] Ahmadifar E., Falahatkar B., Akrami R. (2011). Effects of dietary thymol-carvacrol on growth performance, hematological parameters and tissue composition of juvenile rainbow trout, *Oncorhynchus mykiss*. J. Appl. Ichthyol..

[124] Chishti S., Kaloo Z.A., Sultan P. (2013). Medicinal importance of genus origanum: A review. J. Pharmacogn. Phytother. Acad. J..

[125] Gonçalves R.A., Serradeiro R., Machado M., Costas B., Hunger C., Dias J. (2019). Interactive effects of dietary fishmeal level and plant essential oils supplementation on European sea bass, *Dicentrarchus labrax*: Growth performance, nutrient utilization, and immunological response. J. World Aquac. Soc..

[126] Morel Y., Barouki R. (1999). Repression of gene expression by oxidative stress. Biochem. J..

[127] Ray P.D., Huang B.-W., Tsuji Y. (2012). Reactive oxygen species (ROS) homeostasis and redox regulation in cellular signaling. Cell. Signal..

[128] Halliwell B., Gutteridge J.M. (2015). Free Radicals in Biology and Medicine.

[129] Biller J.D., Takahashi L.S. (2018). Oxidative stress and fish immune system: Phagocytosis and leukocyte respiratory burst activity. An. Da Acad. Bras. De Cienc..

[130] Dawood M.A.O., Koshio S. (2018). Vitamin C supplementation to optimize growth, health and stress resistance in aquatic animals. Rev. Aquac..

[131] Birnie-Gauvin K., Costantini D., Cooke S.J., Willmore W.G. (2017). A comparative and evolutionary approach to oxidative stress in fish: A review. Fish Fish..

[132] Zaki M.A.A., Alabssawy A.N., Nour A.E.A.M., El Basuini M.F., Dawood M.A.O., Alkahtani S., Abdel-Daim M.M. (2020). The impact of stocking density and dietary carbon sources on the growth, oxidative status and stress markers of Nile tilapia (*Oreochromis niloticus*) reared under biofloc conditions. Aquac. Rep..

[133] Tu W.Y., Pohl S., Summpunn P., Hering S., Kerstan S., Harwood C.R. (2012). Comparative analysis of the responses of related pathogenic and environmental bacteria to oxidative stress. Microbiology.

[134] Brewer M.S. (2011). Natural antioxidants: Sources, compounds, mechanisms of action, and potential applications. Compr. Rev. Food Sci. Food Saf..

[135] Embuscado M.E. (2015). Spices and herbs: Natural sources of antioxidants—A mini review. J. Funct. Foods.

[136] Su L., Yin J.J., Charles D., Zhou K., Moore J., Yu L. (2007). Total phenolic contents, chelating capacities, and radical-scavenging properties of black peppercorn, nutmeg, rosehip, cinnamon and oregano leaf. Food Chem..

[137] Abdel-Latif H.M.R., Khalil R.H. (2014). Evaluation of two phytobiotics, *Spirulina platensis* and *Origanum vulgare* extract on growth, serum antioxidant activities and resistance of Nile tilapia (*Oreochromis niloticus*) to pathogenic *Vibrio alginolyticus*. Int. J. Fish. Aquat. Stud..

[138] El-Hawarry W.N., Mohamed R.A., Ibrahim S.A. (2018). Collaborating effects of rearing density and oregano oil supplementation on growth, behavioral and stress response of Nile tilapia (*Oreochromis niloticus*). Egypt. J. Aquat. Res..

[139] Peterson B.C., Bosworth B.G., Li M.H., Beltran R., Santos G.A. (2014). Assessment of a phytogenic feed additive (Digestarom PEP MGE) on growth performance, processing yield, fillet composition, and survival of channel catfish. J. World Aquac. Soc..

[140] Zeppenfeld C.C., Saccol E.M.H., Pês T.S., Salbego J., Koakoski G., dos Santos A.C., Heinzmann B.M., da Cunha M.A., Barcellos L.J.G., Pavanato M.A. (2017). *Aloysia triphylla* essential oil as food additive for *Rhamdia quelen*-stress and antioxidant parameters. Aquac. Nutr..

[141] Hsieh T.J., Wang J.C., Hu C.Y., Li C.T., Kuo C.M., Hsieh S.L. (2008). Effects of rutin from *Toona sinensis* on the immune and physiological responses of white shrimp (*Litopenaeus vannamei*) under *Vibrio alginolyticus* challenge. Fish Shellfish Immunol..

[142] de Freitas Souza C., Baldissera M.D., Bianchini A.E., da Silva E.G., Mourão R.H.V., da Silva L.V.F., Schmidt D., Heinzmann B.M., Baldisserotto B. (2018). Citral and linalool chemotypes of *Lippia alba* essential oil as anesthetics for fish: A detailed physiological analysis of side effects during anesthetic recovery in silver catfish (*Rhamdia quelen*). Fish Physiol. Biochem..

[143] Saccol E.M.H., Londero É.P., Bressan C.A., Salbego J., Gressler L.T., Silva L.V.F., Mourão R.H.V., Oliveira R.B., Llesuy S.F., Baldisserotto B. (2017). Oxidative and biochemical responses in brycon amazonicus anesthetized and sedated with *Myrcia sylvatica* (g. Mey.) dc. And *Curcuma longa* L. Essential oils. Vet. Anaesth. Analg..

[144] Gressler L.T., Riffel A.P.K., Parodi T.V., Saccol E.M.H., Koakoski G., da Costa S.T., Pavanato M.A., Heinzmann B.M., Caron B., Schmidt D. (2014). Silver catfish *Rhamdia quelen* immersion anaesthesia with essential oil of *Aloysia triphylla* (l’hérit) britton or tricaine methanesulfonate: Effect on stress response and antioxidant status. Aquac. Res..

[145] Baldissera M.D., Souza C.F., Júnior G.B., de Vargas A.C., Boligon A.A., de Campos M.M.A., Stefani L.M., Baldisserotto B. (2017). *Melaleuca alternifolia* essential oil enhances the non-specific immune system and prevents oxidative damage in *Rhamdia quelen* experimentally infected by *Aeromonas hydrophila*: Effects on cholinergic and purinergic systems in liver tissue. Fish Shellfish Immunol..

[146] Ali B., Al-Wabel N.A., Shams S., Ahamad A., Khan S.A., Anwar F. (2015). Essential oils used in aromatherapy: A systemic review. Asian Pac. J. Trop. Biomed..

[147] Huang C.-F., Lin S.-S., Liao P.-H., Young S.-C., Yang C.-C. (2008). The immunopharmaceutical effects and mechanisms of herb medicine. Cell. Mol. Immunol..

[148] Peterfalvi A., Miko E., Nagy T., Reger B., Simon D., Miseta A., Czéh B., Szereday L. (2019). Much more than a pleasant scent: A review on essential oils supporting the immune system. Molecules.

[149] Manion C.R., Widder R.M. (2017). Essentials of essential oils. Am. J. Health-Syst. Pharm..

[150] Bousbia N., Abert Vian M., Ferhat M.A., Petitcolas E., Meklati B.Y., Chemat F. (2009). Comparison of two isolation methods for essential oil from rosemary leaves: Hydrodistillation and microwave hydrodiffusion and gravity. Food Chem..

[151] Elyemni M., Louaste B., Nechad I., Elkamli T., Bouia A., Taleb M., Chaouch M., Eloutassi N. (2019). Extraction of essential oils of *Rosmarinus officinalis* L. By two different methods: Hydrodistillation and microwave assisted hydrodistillation. Sci. World J..

[152] Kumar D., Arya V., Kaur R., Bhat Z.A., Gupta V.K., Kumar V. (2012). A review of immunomodulators in the Indian traditional health care system. J. Microbiol. Immunol. Infect..

[153] SaiRam M., Sharma S.K., Ilavazhagan G., Kumar D., Selvamurthy W. (1997). Immunomodulatory effects of nim-76, a volatile fraction from neem oil. J. Ethnopharmacol..

[154] Dalmo R.A., Ingebrigtsen K., Bogwald J. (1997). Non-specific defence mechanisms in fish, with particular reference to the reticuloendothelial system (res). J. Fish Dis..

[155] Cecchini S., Terova G., Caricato G., Saroglia M. (2000). Lysozyme activity in embryos and larvae of sea bass (*Dicentrarchus labrax* L.), spawned by broodstocks fed with vitamin C enriched diets. Bull. Eur. Assoc. Fish Pathol..

[156] Kawakami H., Yamashita H., Sakai M. (2000). Comparative sensitivity of yellowtail *Seriola quinqueradiata* and goldstriped amberjack *S. aureovittata* to photobacterium damsela subsp. Piscicida. J. World Aquac. Soc..

[157] Dossou S., Koshio S., Ishikawa M., Yokoyama S., Dawood M.A.O., El Basuini M.F., Olivier A., Zaineldin A.I. (2018). Growth performance, blood health, antioxidant status and immune response in red sea bream (*Pagrus major*) fed *Aspergillus oryzae* fermented rapeseed meal (RM-KOJI). Fish Shellfish Immunol..

[158] dos Santos M.W., de Brito S.T., de A Prado S., de Oliveira G.C., De Paula C.A., de Melo C.D., Ribeiro A.P.P. (2016). Cinnamon (*Cinnamomum* sp.) inclusion in diets for Nile tilapia submitted to acute hypoxic stress. Fish Shellfish Immunol..

[159] Yilmaz E., Ergün S., Yilmaz S. (2015). Influence of carvacrol on the growth performance, hematological, non-specific immune and serum biochemistry parameters in rainbow trout (*Oncorhynchus mykiss*). Food Nutr. Sci..

[160] Volpatti D., Chiara B., Francesca T., Marco G. (2013). Growth parameters, innate immune response and resistance to Listonella *Vibrio anguillarum* of *Dicentrarchus labrax* fed carvacrol supplemented diets. Aquac. Res..

[161] Sheikhzadeh N., Soltani M., Ebrahimzadeh-Mousavi H.A., Shahbazian N., Norouzi M. (2011). Effects of *Zataria multiflora* and *Eucalyptus globolus* essential oils on haematological parameters and respiratory burst activity in *Cyprinus carpio*. Iran. J. Fish. Sci..

[162] Rattanachaikunsopon P., Phumkhachorn P. (2010). Potential of cinnamon (*Cinnamomum verum*) oil to control streptococcus iniae infection in tilapia (*Oreochromis niloticus*). Fish. Sci..

[163] Soltani M., Sheikhzadeh N., Ebrahimzadeh-Mousavi H., Zargar A. (2010). Effects of *Zataria multiflora* essential oil on innate immune responses of common carp (*Cyprinus carpio*). J. Fish. Aquat. Sci..

[164] Azambuja C.R., Mattiazzi J., Riffel A.P.K., Finamor I.A., Garcia L.d.O., Heldwein C.G., Heinzmann B.M., Baldisserotto B., Pavanato M.A., Llesuy S.F. (2011). Effect of the essential oil of *Lippia alba* on oxidative stress parameters in silver catfish (*Rhamdia quelen*) subjected to transport. Aquaculture.

[165] Awad E., Austin D., Lyndon A.R. (2013). Effect of black cumin seed oil (*Nigella sativa*) and nettle extract (quercetin) on enhancement of immunity in rainbow trout, *Oncorhynchus mykiss* (walbaum). Aquaculture.

[166] Sheikhzadeh N., Soltani M., Mousavi H.E., Khosravi A., Bagheri H., Fathi E., Zargar A. (2009). Effects of *Eucalyptus globules labill* essential oil on some immunological variables of common carp (*Cyprinus carpio*). J. Vet. Res..

[167] Valladão G.M.R., Gallani S.U., Pala G., Jesus R.B., Kotzent S., Costa J.C., Silva T.F.A., Pilarski F. (2017). Practical diets with essential oils of plants activate the complement system and alter the intestinal morphology of Nile tilapia. Aquac. Res..

[168] Sönmez A.Y., Bilen S., Albayrak M., Yılmaz S., Biswas G., Hisar O., Yanık T. (2015). Effects of dietary supplementation of herbal oils containing 1, 8-cineole, carvacrol or pulegone on growth performance, survival, fatty acid composition, and liver and kidney histology of rainbow trout (*Oncorhynchus mykiss*) fingerlings. Turk. J. Fish. Aquat. Sci..

[169] Rafieepour A., Hajirezaee S., Rahimi R. (2019). Dietary oregano extract (*Origanum vulgare* L.) enhances the antioxidant defence in rainbow trout, *Oncorhynchus mykiss* against toxicity induced by organophosphorus pesticide, diazinon. Toxin Rev..

[170] De Moraes França Ferreira P., da Silva Nascimento L., Coelho Dias D., da Veiga Moreira D.M., Lúcia Salaro A., Duca de Freitas M.B., Souza Carneiro A.P., Sampaio Zuanon J.A. (2014). Essential oregano oil as a growth promoter for the yellowtail tetra, *Astyanax altiparanae*. J. World Aquac. Soc..

[171] Dawood M.A.O., El-Salam Metwally A., Elkomy A.H., Gewaily M.S., Abdo S.E., Abdel-Razek M.A.S., Soliman A.A., Amer A.A., Abdel-Razik N.I., Abdel-Latif H.M.R. (2020). The impact of menthol essential oil against inflammation, immunosuppression, and histopathological alterations induced by chlorpyrifos in Nile tilapia. Fish Shellfish Immunol..

