# Supplementary material for: Exploring the Roles of Dietary Herbal Essential Oils in Aquaculture: A Review
Source: Animals (Basel). 2022 Mar 24;12(7):823. doi: 10.3390/ani12070823 (PMC8996993; doi:10.3390/ani12070823)
Supplement: Supplementary file 1 [file animals-12-00823-s001.zip › animals-1319855-supplementary.pdf]

Table S1. Linalool in the essential oil constituents of its natural sources (>10%).

Common name	Botanic name	Oil source	Linalool (%)
Jonquil	*Narcissus jonquilla* L.	Flowers	17.8
Ylang-ylang	*Cananga odorata* J. D. Hook. & T. Thompson f. odorata	Flowers	13.2
Coriander	*Coriandrum sativum* L.	Seeds	59.0–87.5
Coriander	*Coriandrum sativum* L.	Leaves	4.3–17.5
Khella	*Ammi visnaga* L.	Seeds	28.8
Ghandi root	*Homalomena aromatica* Schott.	Rhizomes	62.1
Immortelle	*Helichrysum italicum* subsp. microphyllum	Flowering plant	17.3
Linaloe wood	*Bursera glabrifolia* Humb.	Wood	30.0
Honeysuckle	*Lonicera periclymenum* L	Flowers	75.0
Geranium	*elargonium x asperum* Ehrh. ex Willd	Leaves	13.9
Thyme (linalool CT)	*Thymus vulgaris* L.	Aerial parts	73.6–79.0
Bee balm	*Monarda didyma* L.	Flowering plant	64.5–74.2
Marjoram wild (linalool CT)	*Origanum majorana* L.	Leaves	67.7
Basil (linalool CT)	*Ocimum basilicum* L.	Leaves	53.7-58.3
Mint (bergamot)	*Mentha aquatica* L. var. citrate	Aerial parts	24.9–55.2
Hyssop (linalool CT)	*Hyssopus officinalis* L. var. decumbens Briq.	Aerial parts	48.0-51.7
Basil (hairy)	*Ocimum americanum* L. var. pilosum	Leaves	31.7–50.1
Lavender	*Lavandula angustifolia* Mill.	Flowering tops	25.0–45.0
Lavandin	*Lavandula x intermedia* Emeric ex Loisel	Flowering tops	30.0–38.0
Lavandin Super	*Lavandula hybrida* Reverchon,	Flowering tops	29.4–32.7
Basil (linalool CT)	*Ocimum basilicum* L.	Leaves	34.4
Basil (methyl cinnamate CT)	*Ocimum basilicum* L.	Leaves	17.3–27.3
Clary sage	*Salvia sclarea* L.	Leaves and flowering tops	9.0–19.3
Sage (wild mountain)	*Hemizygia petiolata* Ashby	Aerial parts	15.0
Ho leaf (linalool CT)	*Cinnamomum camphora* Sieb. var. glavescens Hayata	Leaves	66.7–90.6
Rosewood	*Aniba rosaeodora* Ducke	Woods	82.3–90.3
Magnolia leaf	*Michelia alba* DC	Flowers	78.9
Champaca (white)	*Michelia alba* DC	Flowers	76.3
Magnolia flower	*Michelia alba* DC	Flowers	69.9
Rosalina	*Melaleuca ericifolia* Smith	Leaves	35.0–55.0
Niaouli (linalool CT)	*Melaleuca quinquenervia* Cav.	Leaves	23.9
Jasmine sambac	*Jasminum sambac* L.	Flowers	13.9
Tomar seed	*Zanthoxylum armatum* DC	Pericarp of dried fruits	72.0
Neroli	*Citrus x aurantium* L.	Flowers	31.4–54.3
Orange leaf	*Citrus x aurantium* L.	Leaves	42.5
Orange flower	*Citrus x aurantium* L.	Flowers	30.0–32.0

Neroli	*Citrus x aurantium* L.	Flowers	31.4–54.3
Orange flower & leaf water	*Citrus x aurantium* L.	A mixture of flower and leaf water	51.6
Orange leaf (Paraguayan)	*Citrus x aurantium* L.	Leaves	20.8–25.2
Bergamot (expressed)	*Citrus bergamia* Risso & Poit.	Fruit peel, by expression	1.7-20.6
Bergamot (FCF)	*Citrus bergamia* Risso & Poit.	Fruit peel, by expression	4.0-20.0
Ginger lily	*Hedychium coronarium* Koenig	Flowers	29.3
Sanna	*Hedychium spicatum* Sm.	Rhizomes	25.6

Table S2. Myrcene in the essential oil constituents of its natural sources (>10%).

Common name	Botanic name	Oil source	Myrcene (%)
Mastic	*Pistacia lentiscus* L.	Gum resin	0.2-12.3
Pepper (pink)	*Schinus molle* L.	Fruits	50-20.4
Ylang-ylang	*Cananga odorata*	Fruits	24.7
Celery leaf	*Apium graveolens* L.	Leaves and stems	33.6
Parsley (Egyptian)	*Petroselinum crispum* Mill.	Leaves	7.8–23.8
Grindelia	*Grindelia oregana* A. Grey	Aerial parts	14.0-26.0
Lavender cotton	*Santolina chamaecyparissus* L.	Seeds	3.6-15.0
Pteronia	*Pteronia incana* DC	Aerial parts	10.3
Tansy (blue)	*Tanacetum annuum* L.	Aerial parts of flowering plant	1.1-13.8
Field wormwood	*Artemisia campestris*	Wood	11.2
Jambu flower	*Acmella oleracea*	Flowers	11.8
Sudanese frankincense	*Boswellia sacra* (a-pinene CT)	Gum resin	20.7
Hemp	*Cannabis sativa* L.	Flowering tops	21.2-31.1
Juniperberry	*Juniperus communis* L	Berries	22.0
Rosemary (β-Myrcene CT)	*Rosmarinus officinalis* L.	Aerial parts	19.5–52.1
Wild thyme	*Thymus serpyllum*	Flowering herb	39.1
Hop	*Humulus lupulus* L	Inflorescence	25.4
Bay "West Indian"	*Pimenta racemosa* var. racemosa (Miller) J. Moore	Leaves	6.4–25.0
Bay "Anise"	*Pimenta racemosa* var. racemosa (Miller) J. Moore	Leaves	12.8
Myrtle (honey)	*Melaleuca teretifolia* Endl.	Leaves	10.9
Pine (white)	*Pinus strobus* L.	Needles (leaves) and branches	4.7-13.1
African bluegrass	*Cymbopogon validus* Stapf	Leaves	15.4–20.2
Lemongrass (West India)	*Andropogon citratus* DC	Leaves	5.6–19.2
Cape May	*Coleonema album* (Thunb.) Bartl. & J.C. Wendl.	Leaves and stem	43.8
Pepper (Sichuan)	*Zanthoxylum piperitum* DC	Fruits	16.4

Table S3. Eucalyptol (1,8-cineole) in the essential oil constituents of its natural sources (>10%).

Common name	Botanic name	Oil source	Eucalyptol (%)
Lanyana	*Artemisia afra* von Jacquin	Leaves and stems	19.1
Rambiazana	*Helichrysum gymnocephalum* Humbert	Leaves	47.4
Sage (African wild)	*Tarchonanthus camphoratus* L.	Leaves and flowers	15.5
Southernwood	*Artemisia abrotanum* L.	Aerial parts	18.6
Wormwood	*Artemisia annua* L.	Flowering plant	14.7
Saro	*Cinnamosma fragrans* Baill.	Leaves	46.0–53.0
Basil (holy)	*Ocimum tenuiflorum* L.	Leaves	12.6–16.5
Hyssop	*Hyssopus officinalis* L. var. decumbens Briq.	Aerial parts	12.3–14.9
Lavandin	*Lavandin Abrialis*	Flowering tops	6.0-11.0
Lavandin	*Lavandin Grosso*	Flowering tops	5.2-10.2
Lavender (Spanish)	*Lavandula stoechas* L. ssp. stoechas	Flowering tops	3.6–14.5
Lavender (spike)	*Lavandula latifolia* Medic.	Flowering tops	27.2–43.1
Marjoram (Spanish)	*Thymus mastichina* L. ssp. mastichina	Aerial parts of flowering plant	45.1–58.6
Rosemary (Borneol)	*Rosmarinus officinalis* L.	Aerial parts	20
Rosemary (Bornyl acetate CT)	*Rosmarinus officinalis* L.	Aerial parts	6.8-13.6
Rosemary (Camphor CT)	*Rosmarinus officinalis* L.	Aerial parts	17-22.5
Rosemary (1,8-Cineole CT)	*Rosmarinus officinalis* L.	Aerial parts	39.0-57.7
Rosemary (α-Pinene CT)	*Rosmarinus officinalis* L.	Aerial parts	15.0-25.1
Sage (Dalmatian)	*Salvia officinalis* L.	Leaves	1.8-21.7
Sage (Greek)	*Salvia fruticosa* Mill.	Leaves	59.0
Sage (Spanish)	*Salvia lavandulifolia* Vahl.	Flowering tops	12.0-40.3
Sage (white)	*Salvia apiana* Jeps.	Leaves	68.4
Ho leaf (Chinese)	*Cinnamomum camphora* L.	Leaves	50.0
Ho leaf (Madagascan)	*Cinnamomum camphora* L.	Leaves	56.7-63.7
Laurel or Bay leaf	*Laurus nobilis* L.	freshly picked leaves	38.1–43.5
Sugandha	*Cinnamomum cecidodaphne* Meisn.	Fruits	13.1
Boldo	*Peumus boldus* Molina	Dried leaves	21.1
Cajuput	*Melaleuca cajuputi* Powell	Leaves and twigs	41.1–70.8
Eucalyptus	*Eucalyptus camaldulensis* (cineole CT)	Leaves	46.9–83.7
Eucalyptus	*Eucalyptus globulus*	Leaves	65.4–83.9
Eucalyptus	*Eucalyptus maidenii*	Leaves	76.8
Eucalyptus	*Eucalyptus plenissima*	Leaves	85.0–95.0
Eucalyptus	*Eucalyptus polybractea*	Leaves	88.7–91.9
Eucalyptus	*Eucalyptus radiata*	Leaves	60.4–64.5
Eucalyptus	*Eucalyptus smithii*	Leaves	77.5
Eucalyptus	*Eucalyptus macarthurii* H. Deane & Maiden	Leaves	28.9–29.0

Eucalyptus	*Eucalyptus polybractea* R. T. Baker	Leaves	16.1
Fragonia	*Agonis fragrans* J.R.Wheeler & N.G.Marchant	Leaves	31.0-33.0
Myrtle	*Myrtus communis* L.	Leaves	18.9–37.5
Niaouli	*Melaleuca quinquenervia* Cav.	Leaves	55.0–65.0
Rosalina	*Melaleuca ericifolia* Smith	Leaves	18.0–26.0
White kunzea	*Kunzea ambigua* (Sm.) Druce	Leaves	14.8
Damiana	*Turnera diffusa* Willd. var. aphrodisiaca Urb.	Leaves	11.4
Chaste tree	*Vitex agnus* castus L.	Leaves	15.6–35.2
Chaste tree	*Vitex agnus* castus L.	Seeds	8.4-23.3
Oregano (Mexican)	*Lippia berlandieri*	Dried aerial parts of flowering plant	1.8–14.0
Cardamon	*Elettaria cardamomum* L.	Seeds	26.5–44.6%
Cardamon (black)	*Amomum subulatum* Roxb.	Seeds	61.3
Galangal (greater)	*Alpinia galanga* L.	Rhizomes	30.2–33.6
Galangal (lesser)	*Alpinia officinarum* Hance	Rhizomes	49.6
Sanna	*Hedychium spicatum* Sm.	Rhizomes	44.3
Zedoary Indian (hydrodistilled)	*Curcuma zedoaria* Roscoe	Rhizomes	15.9

Table S4. β- caryophyllene in the essential oil constituents of its natural sources (>10%).

Common name	Botanic name	Oil source	β- caryophyllene (%)
Ylang-ylang	*Cananga odorata*	Flowers	11.2
Cananga	*Cananga odorata* (Lam.)	Flowers	38.2
Mugwort	*Artemisia vulgaris* L.	Aerial parts	10.6
Hemp	*Cannabis sativa* L.	Flowering tops	13.7-19.4
Piri-piri	*Cyperus articulatus* L.	Rhizomes	4.6–13.7
Camphor	*Dryobalanops aromatica* Gaertn	Woods	18.1
Fenugreek	*Trigonella foenum*-graecum L.	Seeds	14.6
Copaiba	*Copaifera langsdorfii*	Balsam from wood	53.3
Blackcurrant	*Ribes nigrum* L.	Flower buds	9.0-14.0
Savory	*Satureia montana* L.	Dried aerial parts	0-13.6
Thyme	*Thymus serpyllum*	Aerial parts	6.0–11.2
Melissa	*Melissa officinalis* L.	Aerial parts	0.3-19.1
Catnip	*Nepeta cataria* L.	Aerial parts	6.0-24.6
Basil	*Ocimum americanum* L.	Leaves	4.3-10.0
Cangerana	*Cabralea cangerana* Saldanha	Woods	28.6
Myrtle	*Myrica gale* L.	Leaves	11.0
Clove	*Syzygium aromaticum* (L.) Merill et L.M. Perry	Flower buds and stems	0.6-12.4%
Pine	*Pinus nigra* J. F. X Arnold	needles leave	5.3–11.8
Pepper	*Piper nigrum* L.	Fruits	9.4–30.9
Fern	*Comptonia peregrina* (L.) J. M. Coult.	Leaves	24.5
Inula	*Pilocarpus microphyllus*	Flowering tops	23.9-40.6
Lantana	*Lantana camara* L.	Flowering tops	12.0
